# Swiping colors in virtual reality: How stable are color category borders?

**DOI:** 10.1167/jov.26.4.9

**Published:** 2026-04-16

**Authors:** Avi M. Aizenman, Zoe R. Goll, Raquel Gil Rodriguez, Karl R. Gegenfurtner

**Affiliations:** 1 Psychology Department, Giessen University, Giessen, Hessen, Germany

**Keywords:** color categories, virtual reality, range effect

## Abstract

Human color perception involves a tradeoff between our ability to discriminate millions of continuous hues and our reliance on a few discrete linguistic categories. Although some theories suggest these category boundaries are fixed perceptual anchors, others propose that judgments adapt dynamically to the statistical distribution of recent stimuli, known as the range effect. To test the stability of these boundaries, we adapted a fast-paced "match-to-sample" paradigm from animal learning into an immersive VR videogame. Participants used colored sabers to strike incoming cubes, matching saber color to the cube's stripe. We tested both the blue-green boundary (aligned with low-level cone mechanisms) and the pink-purple boundary (off-axis), using hue sets equated for discriminability. After establishing baseline category borders using psychometric functions, we shifted the range of tested colors toward one category endpoint to determine if the internal border remained stable or shifted with the stimulus distribution. Across four experiments, results consistently revealed a partial shift. Rather than remaining invariant, category borders shifted systematically in the direction of the stimulus range shift. Further manipulations demonstrated that this partial shift was unaffected by the proportion of responses and occurred even when using hues which don't contain a category boundary. These findings indicate that under rapid decision-making conditions, observers’ judgments are strongly influenced by the statistical structure of the immediate stimulus set, with stable categorical anchors playing a more limited role. This suggests a limited role for linguistic color categories in active, matching-based tasks, where observers likely prioritize automatic statistical adaptation over fixed categorical distinctions.

## Introduction

Humans can discriminate between one thousand and two million different colors, depending on experimental conditions (Linhares, Pinto, & Nascimento, 2008; Pointer & Attridge, 1998; Koenderink, van Doorn, & Gegenfurtner, 2018; Koenderink, van Doorn, & Gegenfurtner, 2019; Koenderink, van Doorn, & Gegenfurtner, 2020; Krauskopf & Gegenfurtner, 1992). Despite this enormous capacity to discriminate hue, lightness, and saturation, we typically use only a few discrete color terms to describe and communicate (Berlin & Kay, 1969). This reduces a vast continuum to a select set of color categories. The clear distinction between our continuous color perception and our use of color categories for communication has sparked extensive research on categorical effects for color perception.

Much of this work has focused on the relationship between perception and language (see Harnad, 1987). However, recent findings suggest that categorical effects in humans may be less fixed than once assumed, varying with attention, task demands, and observer experience (for a recent review, see Witzel & Gegenfurtner, 2018). To revisit these issues, we applied a fast decision paradigm inspired by match-to-sample tasks widely used in the animal literature, where categorical behavior is probed through rapid repeated choices. Our goal was to adapt this logic to human observers in an engaging format that enables large numbers of decisions in a short time, providing a sensitive test of category border stability.

Categorical perception of color refers to the tendency for people to be faster and more accurate at discriminating colors that belong to different categories (such as “blue” and “green”) compared to those within the same category (such as two different shades of “blue”). This suggests that the presence of a category boundary between two colors enhances perceptual sensitivity to differences across categories (Bornstein & Korda, 1984; Daoutis, Pilling, & Davies, 2006; Gilbert, Regier, Kay, & Ivry, 2006; Holmes, Franklin, Clifford, & Davies, 2009; Witzel & Gegenfurtner, 2011). Furthermore, language specific category effects have been observed (Thierry, Athanasopoulos, Wiggett, Dering, & Kuipers, 2009; Winawer et al., 2007) and similar facilitation can be induced by training observers on novel categories (Özgen, 2004; Özgen & Davies, 2002; Zhou et al., 2010).

### The blue-green boundary and low level color mechanisms

Many well-known categorical effects concern the blue–green boundary. Importantly, this boundary coincides with the L–M axis of second-stage cone-opponent mechanisms (Derrington, Krauskopf, & Lennie, 1984; Krauskopf, Williams, & Heeley, 1982), where chromatic sensitivity is naturally enhanced. Because much of the empirical literature focuses on this region (Bornstein & Korda, 1984; Drivonikou et al., 2007; Gilbert et al., 2006; Özgen & Davies, 2002; Roberson, Hanley, & Pak, 2009), it is difficult to determine whether observed effects reflect true categorical structure or differences in low-level discriminability. When discriminability is equated across hues, category effects at the blue–green boundary often disappear (He, Witzel, Forder, Clifford, & Franklin, 2014; Lindsey et al., 2010; Witzel & Gegenfurtner, 2013). This raises the question of whether category effects observed here generalize to non-cardinal regions of color space.

### Training and attention

If color categories reflect a genuinely perceptual boundary, they should be robust to experience and training. Yet trained observers often show reduced or absent categorical effects, even though they perform the basic discrimination task faster and more accurately (Witzel & Gegenfurtner, 2015). ERP studies similarly indicate that categorical effects emerge primarily at later, post-perceptual stages associated with attention (He et al., 2014). Together, these findings suggest that at least some category effects may arise from attentional or decisional processes rather than from early sensory mechanisms.

### Category stability in non-human species

Color categorization has also been investigated in non-human species using match-to-sample paradigms. Several species, including songbirds, chicks, goldfish, and non-human primates, show apparently stable category boundaries (Caves et al., 2018; Zipple et al., 2019; Jones, Osorio, & Baddeley, 2001; Poralla & Neumeyer, 2006; Davidoff & Fagot, 2010; Fagot et al., 2006; Matsuno, Kawai, & Matsuzawa, 2004; Sandell, Gross, & Bornstein, 1979).

Classic work by Wright and Cumming (1971) used a match-to-sample task to test the invariance of color category boundaries in pigeons. After training pigeons to match colors to one of three samples, they introduced new colors to see which sample the pigeons would choose. These measurements reveal the hues where pigeons switch from matching one color to another. When the experiment was repeated with different training colors, pigeons showed remarkably consistent crossover points across experiments, despite the shift in training hues between the first and second study. This was interpreted as a sign that pigeons have a categorical perception of color.

However, recent work by Garside, Chang, Selwyn, and Conway (2025) questions such findings in animals. They used a match-to-sample task to evaluate biases in color perception. Their monkeys showed no consensus color categories. When they applied their paradigm to humans, they uncovered several color categories, but these were not in alignment with previous results from the World Color Survey (Lindsay & Brown, 2025). Their results suggest that color categories in humans might depend on language, but also that color categories might be more complex than assumed based on previous results.

### Statistical adaptation and the range effect

Beyond issues of discriminability and retinal mechanisms, a separate body of work suggests that perceptual judgments, including those of color, are heavily influenced by the statistics of the stimulus set—a phenomenon known as the *range effect*. Range effects have been observed across various perceptual domains, including timing estimation (Jazayeri & Shadlen, 2010; Mamassian & Landy, 2010), estimates of size (Hollingworth, 1910), shape and grey value (Huttenlocher, Hedges, & Vevea, 2000), line length (Ashourian & Loewenstein, 2011; Duffy, Huttenlocher, Hedges, & Crawford, 2010), and vibrotactile perception (Aizenman, Gaff & Voudouris, 2025). This effects refers to a systematic bias in responses toward the mean or central tendency of the underlying stimulus distribution, as observers implicitly adapt to the range of stimuli tested (Parducci, 1965).

In color perception, range effects have been shown for hue (Olkkonen, McCarthy, & Allred, 2014; Saarela, Niemi, & Olkkonen, 2023). Saarela et al.’s work also shows short-term effects such as serial dependence independently contribute alongside range effects to bias perceptual judgments. This suggests that perceptual judgments of color are not absolute, but are instead normalized to the set of colors recently or currently experienced. Bayesian models provide a natural account of these findings: perceptual estimates reflect a combination of sensory evidence and expectations about stimulus prevalence (Ashourian & Loewenstein, 2011; Jazayeri & Shadlen, 2010). Related work on ensemble color perception suggests that these are strongly affected by the range effect, but that linguistic categories contribute only minimally (Virtanen, Saarela, & Olkkonen, 2024). Given these findings, the central question arises whether linguistic color-category boundaries are fixed and invariant or whether they shift dynamically with changes in the relative frequency and range of hues presented in the stimulus set.

### Rationale for the present study

In humans, the contribution of category boundaries versus statistical adaptation has not been directly tested in a fast, repeated-decision paradigm analogous to the animal literature. The present study was designed to fill this gap. We adapted a match-to-sample task to a virtual-reality (VR) environment modeled after a Beat Saber-style game. VR provides several advantages for this purpose: it enables immersive, sustained engagement; it allows rapid sequences of forced-choice decisions; and, with proper calibration (Díaz-Barrancas, Gil-Rodríquez, Aizenman, Bayer, & Gegenfurtner, 2023; Gil Rodríguez et al., 2022), it affords precise chromatic control comparable to traditional laboratory displays. The active nature of the task also parallels the structure of animal match-to-sample experiments, making it possible to test category border stability under conditions that encourage rapid, intuitive responses.

To isolate category effects from discriminability differences, we used hue sets defined by multiples of JNDs from Witzel and Gegenfurtner (2013), ensuring that neighboring test colors were equally discriminable. We tested two category boundaries—blue–green, aligned with the L–M axis, and pink–purple, which lies off-axis—allowing us to compare regions with and without known low-level effects.

If category borders are robust linguistic anchors, they should remain invariant across shifts in the hue range. If responses reflect direct comparison between test and choice stimuli, category borders should shift fully with the stimulus set. If judgments are influenced by statistical adaptation, we would expect partial shifts toward the mean of the tested range.

### Outline

Across four experiments, we manipulated the range of hues presented, the prevalence of category-consistent stimuli, the similarity between test and choice stimuli, and the presence or absence of a category boundary. In every case, observers’ category borders shifted partially toward the center of the stimulus distribution. This pattern of results indicates that under rapid decision-making conditions, observers rely primarily on the statistical structure of the stimulus set rather than on stable categorical anchors.

## Experiment 1: Gamified category borders

As a starting point, Experiment 1 measured the point of subjective equivalence (PSE) defining the color category border in our VR match-to-sample task. By systematically shifting the range of tested hues across blocks, we asked whether the estimated border would remain stable—as predicted by fixed category accounts—or shift in accordance with the underlying range of hues tested.

### Methods

#### Apparatus

Observers were presented with a color-swiping, match-to-sample game implemented within an immersive virtual environment akin to the popular BeatSaber video game. Observers were instructed to swipe through a colored block with a pair of colored sabers, one blue and one green. The colored block is oriented horizontally or vertically, and observers swipe the colored block orthogonally with the saber color that best matches the current block color. Although there is a steady stream of colored blocks approaching the observer, each colored block is presented to the user one at a time. The color-swiping game was presented using the HTC Vive Pro Eye headset. The headset includes two OLED screens, one for each eye, with a resolution of 1440 × 1600 pixels per eye. The games were run on a PC with Windows 10 64-bit operating system, AMD Ryzen Threadripper 3990 × 64-core processor with 2.9 GHz, 256 GB RAM, and an NVIDIA GeForce RTX 3090 graphics card. The experiment was developed using Unity (version 2019.3.8f1), and the game maintained a frame rate of approximately 90 Hz.

#### Participants

Twenty participants (eight males, twelve females), aged 22–39, took part in the experiment. All participants passed the Ishihara color vision test (Ishihara, 1997), confirming normal color vision. The experimental protocol was approved by the Institutional Review Board at our university in accordance with the Declaration of Helsinki (Ethics approval no. LEK 2020-0015). Participants signed informed consent forms before participating.

#### Stimuli

**Figure 1.**
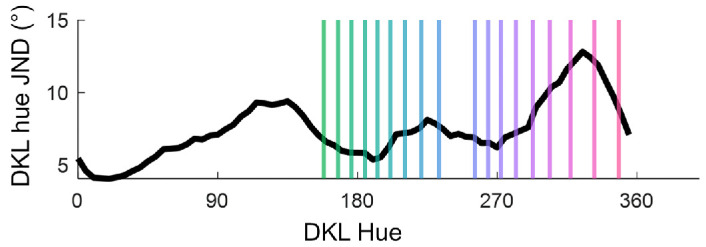
Blue-green and pink-purple hues were selected for the experiment based on data from Witzel and Gegenfurtner (2013). The black curve shows the original data collected plotting JND over the hue circle. Vertical lines represent blue-green and pink-purple hues selected for the current experiment. Neighboring blue-green hues are separated by 1.5 JNDs and pink-purple by 1.3 JNDs.

We used the DKL color space introduced by Krauskopf and colleagues (Krauskopf, Williams & Heeley, 1982; Derrington et al., 1984; see also Hansen & Gegenfurtner, 2013). Hues were selected to smoothly vary in fixed multiples of the discrimination threshold between adjacent test colors. We sampled stimuli around the best estimation of the category borders for blue-green and pink-purple hues while also spanning prototypical examples for each category. Previous work by Witzel and Gegenfurtner (2013) characterized discrimination thresholds around the hue circle and estimated the category border for each color category. To measure this, (Witzel & Gegenfurtner, 2013) presented observers with four colored circles and asked observers to select which one was different. This paradigm is useful for measuring JNDs between colors. The black curve in Figure 1 shows the discrimination thresholds estimated from this paradigm. These original data (cf., Figure 3, (Witzel & Gegenfurtner, 2013)) have been smoothed with a Loess filter with a window span of six values revealing systematic variations in discrimination thresholds across hues.

Pilot testing with our setup estimates the blue-green and pink-purple category borders at 193° and 293°, respectively. Using the JND thresholds shown in Figure 1, eight test hues were selected around these borders, ensuring they spanned the boundary and included prototypical examples. Neighboring hues were separated by 1.5 JNDs for blue-green and 1.3 JNDs for pink-purple hues. The slightly smaller spacing for pink-purple hues was necessary to fit all stimuli into the smaller range for those colors. Figure 1 shows the distribution of the hues as vertical lines, and Supplementary Appendix Table A1 provides stimulus details for the selected hues.

**Figure 2.**
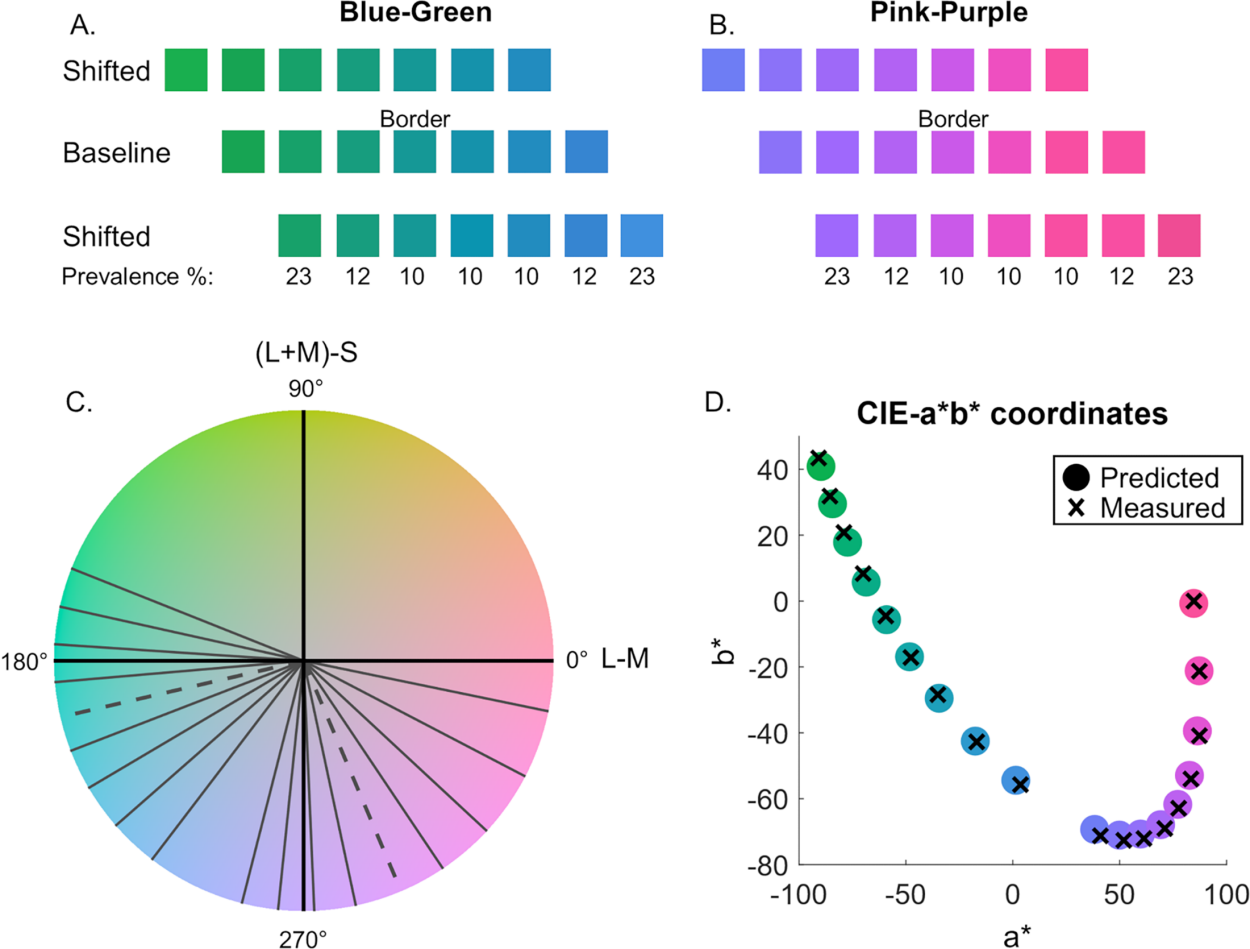
(**A** and **B**) The three sets of hues tested for blue-green and pink-purple. Shifted hues comprise six of the same colors as the baseline set, and the seventh hue shifts the overall set of colors to be more green or blue and pink or purple relative to the baseline. The center color in the baseline condition represents the category border. For blue-green hues, there are 1.5 JNDs between neighboring hues, and 1.3 JNDs for pink/purple hues. Under the bottom row of hues, the prevalence of each hue is shown. The same prevalence scheme was applied to each set of hues. (**C**) The isoluminant plane of the DKL color space. The hues tested are shown in gray, and a dotted line represents the category border. (**D**) The cross shows colors measured by the spectroradiometer, and the filled circles represent the predicted values from the color characterization procedure. Data are represented in CIEL*a*b*, specifically the a*-b* 2D plane.

From the nine selected hues, we created three sets of colors; a baseline set, and two ‘shifted’ sets as shown in Figure 2A for blue-green and Figure 2B for pink-purple. The baseline set included three hues on each side of the blue-green and pink-purple borders. The “blue-shifted” and “green-shifted” sets were created by removing one hue from the baseline set and adding another blue or green hue. This resulted in six shared hues and one shifted hue in the “shifted” sets. Figure 2C shows how the selected colors are distributed along the hue circle in DKL color space.

Unity relies on RGB values to define the color of objects. We used the dkl2rgb function from the Python-based Psychopy software (Peirce et al., 2019) to convert DKL color values to RGB. These values were applied to objects using Unity's “Unlit” shader. Unlike standard shaders that interact with scene lighting through shading and highlights, the Unlit shader renders a constant color regardless of light sources. This eliminates the influence of shadows and specular highlights on the saber and cube colors, ensuring the chromaticity remains invariant. To maintain spatial depth and immersion, the surrounding environment (walls and floors) was rendered using Unity's standard material, which supports lighting and shadow effects, thereby providing the necessary structural cues for a three-dimensional scene.

The CIE1931 chromaticity coordinates and luminance of the HTC Vive Pro Eye's display primaries were: R = (0.6556, 0.3435, 33.8211), G = (0.2195, 0.7145, 87.6361), B = (0.1461, 0.0387, 6.2931), and W = (0.3003, 0.3311, 121.7036). The HMD displays were characterized using the methods we developed for VR headsets (Díaz-Barrancas et al., 2023; Gil Rodríguez et al., 2022; Toscani et al., 2019). We began by taking measurements for each color channel (R, G, and B) separately and for the three color channels together (from black to white). These measurements were taken using the CS-2000A spectroradiometer (Konica Minolta, Tokyo, Japan), which provides measurements of XYZ for characterization of the HMD device*.* The previous methods describe a colorimetric characterization procedure that defines the relationship between the RGB values of the engine and the XYZ values measured from the display by the spectroradiometer. This process transforms RGB values from the game engine into predicted displayed values based on the characterization procedure. Figure 2D shows the 18 different chromaticities represented in CIEL*a*b* color space, specifically the a*-b* 2D plane. The crosses show the measured values from the CS-2000A spectroradiometer, and the colored circles show the predicted values based on the colorimetric characterization procedure for the hues tested in the current study. In addition, we calculated the CIEDE2000 values (Sharma, Wu, & Dalal, 2005) between the estimated and predicted values, with a mean of 0.680 and standard deviation of 0.347. The maximum value was 1.257, with only four values exceeding 1. This suggests the difference between the measured and predicted colors is perceptually equivalent.

#### Experiment design

The experiment was conducted over two sessions during two days, one focusing on blue-green hues and the other on pink-purple hues. Each session consisted of three blocks; one baseline, and two shifted sessions. Blocks lasted 16 minutes each and the presentation order of the blocks was counterbalanced using a Latin Square Design. At the beginning of the experimental session, participants placed the HMD on their head. They adjusted the headset until it reached a comfortable position that enabled a full field of view. They adjusted the separation between the left and right displays to match their inter-ocular distance.

Each block commenced with the participants in an empty room, holding a virtual “saber” in each hand. The color of the sabers was set as the endpoint hue of each set of colors tested (as shown in Figures 2A and 2B). Participants were shown a series of dark grey cubes with a colored stripe that approached gradually from a distance, as shown in Figure 3. Participants were instructed to swipe the cubes with the saber that best matched the hue of the colored stripe on the cube. Additionally, they were asked to swipe the colored stripe in a direction perpendicular to the cube. During the first minute of gameplay, participants were only shown cubes with colored stripes that matched the hue of the sabers. After this first minute, the rest of the hues from the current set were introduced. The endpoint hues (those farthest from the border) were presented at a higher prevalence to ensure that prototypical hues for each category were enforced. Cubes that matched the saber color were presented with a prevalence of 23% and the adjacent hue 12%, compared to the border hue and border-adjacent hues, which were each presented at a prevalence of 10%, as shown in Figure 2.

**Figure 3.** A brief video of the experiment. Observers held a colored “saber” in each hand and swiped approaching cubes. Observers were instructed to swipe each cube with the saber that best matched the colored stripe and to swipe perpendicular to the colored stripe. For example, the nearest cube in the schematic should be swiped from the right to the left with the blue saber. If the swipe direction and saber color were correct, the cube would emit fireworks and disappear.

For cubes that contained the border hue or the border-adjacent hues, if the participant hit the cube with either saber, the cube would disappear, accompanied by a release of fireworks. If the swipe was in the correct orientation, a swiping noise played. For cubes that matched the saber color, participants had to swipe with the correctly colored saber. Otherwise, if they swiped with the wrong saber, the cube would continue approaching, unchanged. New cubes were generated at a consistent interval every ˜1.1 seconds. Musical accompaniment was provided to enhance the immersive experience for the observers while they performed the task. The musical selections were curated to maintain a tempo of 105 beats per minute in concordance with the pace of the cubes. Observers made ˜55 classifications per minute, and ˜840 classifications in total for each block. Halfway through the session, the saber colors were swapped between the left and right hands. A text prompt alerted participants to this change, which served to counterbalance the task and eliminate potential response biases associated with handedness

#### Statistical analyses

All statistical analyses were conducted in JASP (JASP Team, 2023). A one-way repeated-measures analysis of variance (ANOVA) with the factor condition (shifted or baseline) was computed for behavioral metrics. If there was a main effect, post-hoc *t*-tests evaluated differences between conditions, and in order to avoid the multiple comparison problem, we report the adjusted *p*-value using a Holm correction.

### Results

**Figure 4.**
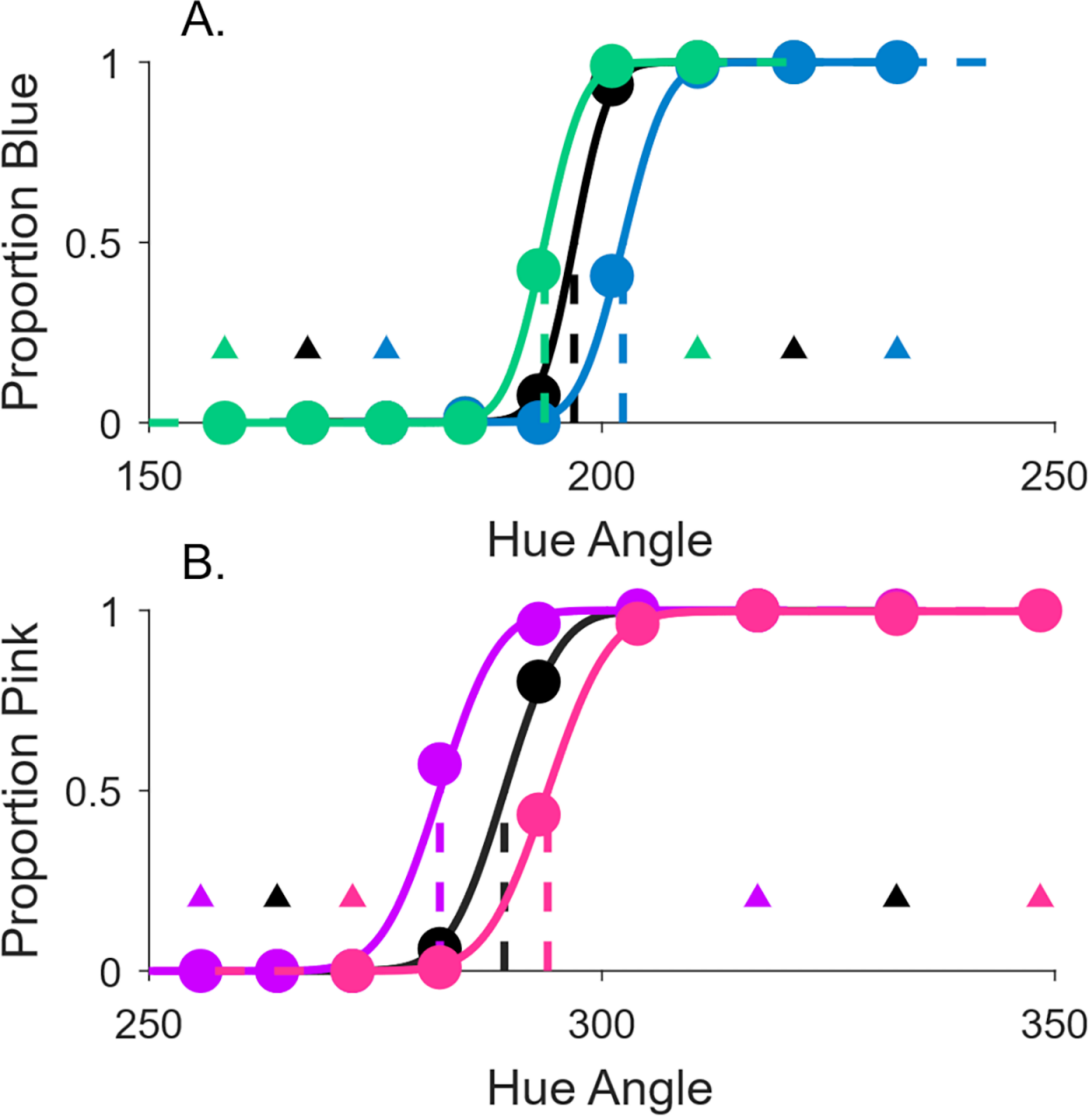
Psychometric fits for data from a single representative observer, and average PSE values for all experiments. Psychometric fits for blue-green (**A**) and pink-purple data (**B**). The horizontal axis in both plots shows hue angle (in DKL), while the proportion of cubes swiped with the blue saber (**A**) or the pink saber (**B**) is depicted on the vertical axis. Circular data points represent the hues tested, and the small triangles show the saber hues for that particular condition. Data are fit with a cumulative Gaussian function, and a vertical line marks the 50% point of PSE. Three separate psychometric functions were fit for data from each of the three blocks/sets of hues tested for blue-green and pink-purple. The black line shows data from the unshifted baseline condition.

We compared the proportion of swipe responses for each color set to investigate the stability of color category borders. We calculated separate psychometric functions for each block of trials using the Psignifit toolbox (version 4 for Matlab (Schütt, Harmeling, Macke, & Wichmann, 2016)). We used a cumulative Gaussian distribution with four parameters which included the PSE, the σ of the function, guess rate, and lapse rate. Figures 4A and 4B show the psychometric curves for a representative observer for blue-green and pink-purple data. This represents the proportion of swipe responses made using either the blue or pink saber as a function of hue. The vertical line on each psychometric function indicates the point of subjective equality. The PSE represents the hue at which observers choose each saber with equal frequency; the category border between blue-green or pink-purple. If the category border was perfectly stable, there would be no difference between the PSE for the baseline condition and the two shifted conditions; the psychometric functions should overlap. Alternatively, if observers are matching the saber hues to the cube, responses could show a displacement consistent with a range effect; the PSEs may shift in the same direction as the colors tested. In the blue-green condition, this would present as a larger PSE for the blue-shifted hues, and smaller PSE for the green-shifted hues relative to the baseline PSE. Figures 4A and 4B show a pattern of shifted PSEs; the three psychometric curves do not overlap, but are distinct, both for blue-green and pink-purple hues. The category border shows a systematic shift toward the direction of the shift in the hues tested, both for blue-green and pink-purple data for this observer.

**Figure 5.**
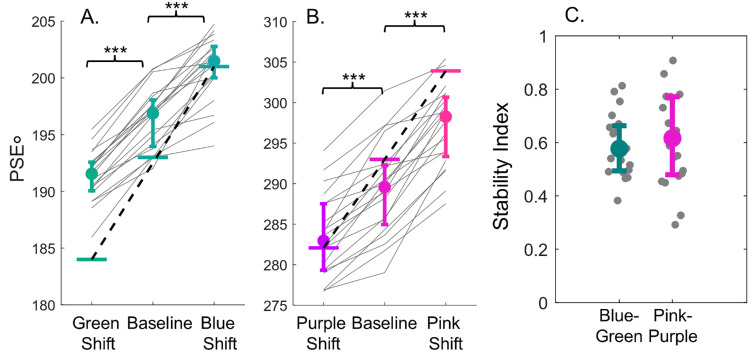
Median PSE for blue-green (**A**) and pink-purple (**B**) hues, where the PSE represents the category border/decision boundary. Circular markers represent the overall average across observers while error bars represent the 25th and 75th percentile. The horizontal line closest to the median represents the center hue for that set of colors tested. Each observer's data is represented by a light gray line. If observers shifted their category border commensurate to the different hues tested, the median and this marker would align. Additionally, the data would fall along the dashed black line, which represents a full shift of the category border with respect to the hues tested. Error bars show the interquartile range. (**C**) Stability Index where the vertical axis shows the ratio of the difference in the PSEs and the difference in center hues for the shifted conditions. A value of 0.0 would correspond to perfectly stable borders, because the PSE remained the same across the shifted conditions. Circular markers represent the overall median across observers, and error bars represent the interquartile range. *p < 0.05; **p < 0.01; ***p < 0.001.

Figures 5A and 5B show the average PSE for the different conditions for blue-green and pink-purple data. Median PSE is represented by the filled circles, and the horizontal dotted line represents the center hue for the colors tested. The two solid horizontal lines represent the saber hues. We compared the shift of the PSEs by running separate one-way ANOVAs for the blue-green [*F*(2, 38) = 240, *p* < 0.0001, $\eta_{p}^{2}$= 0.93] and pink-purple data [*F*(2, 38) = 156, *p* < 0.0001, $\eta_{p}^{2}$ = 0.892], both of which showed a main effect. Post-hoc tests confirmed significant differences between the PSEs for all conditions for blue-green and pink-purple data (all *p* < 0.0001). The pink-purple data show a wider spread and greater variability between observers than the blue-green data, but the bias is persistent across both sets of hues.

How stable are the PSEs relative to the shift in the hues tested? We examined the relationship between the difference in category borders for the shifted stimuli and the shift in the colors tested. For each observer, we computed a stability index, which is the ratio of the difference between the PSEs in the shifted condition to the difference between the center hue of the shifted colors. If category borders remained stable, we would anticipate the differences in PSEs between the shifted conditions to be 0, resulting in a stability index of 0. Alternatively, if observers based their responses on the underlying stimulus distribution, the difference in PSEs would match the difference in stimuli between the shifted conditions. In this scenario, the stability index would be 1.0, showing a full range effect. The results of this analysis are shown in Figure 5C. On average, observers’ stability indices are falling halfway between perfectly stable borders, and a full range effect. Observers show a shift in their category border, which falls short of a full shift. This suggests category borders are shifting but that this falls short of what we would expect to see if observers only based their responses on the underlying stimulus distribution.

### Summary

The findings from the initial experiment demonstrate that color category borders are influenced by the set of hues presented. We originally hypothesized two outcomes: either the color category borders would remain stable regardless of the shift in hues, or they would fully shift in alignment with the hue changes, reflecting a range effect. Contrary to these predictions, our results revealed a partial shift in the responses, indicating that category borders are not completely stable, but somewhat responsive to the changes in stimulus range. This held for both types of category border, blue-green and pink-purple. While the blue-green border closely aligns with the second-stage L-M mechanism, this is less the case for the pink-purple border. The similarity in the results suggests that low-level mechanisms do not play a major role here.

In this experiment, the endpoints of the hue range were presented more frequently to emphasize prototypical colors, a method aligned with the match-to-sample approach often used in animal studies Wright and Cumming (1971). This implies that the shifted condition presented a greater proportion of one color category than the other, leading to a potential imbalance in the responses during each condition. Observers might adjust their response criteria to achieve an equal number of responses for each category, thereby also using each saber equally often. Could this drive the partial shift in their responses? In a follow-up experiment, we test the stability of the category border when the proportion of hues shown from each category is equalized.

## Experiment 2: Balanced hues

In Experiment 2, we investigate whether the partial shift in the PSE persists when the proportion of cubes from each color category is balanced. If the shift is driven to the greater prevalence of colors from one category, balancing the hues should equalize the response proportions, and eliminate the shift effect. Alternatively, if the shift effect remains, this would suggest that the shift in category borders is robust to hue prevalence.

### Methods

#### Observers

Ten new observers (seven female, three male, aged 19–34), all naive to the experiment's purpose, participated. All had normal color vision as confirmed by the Ishihara color vision test (Ishihara, 1997). The experimental setup was identical to the first experiment, with key differences noted below.

#### Experimental design

**Figure 6.**
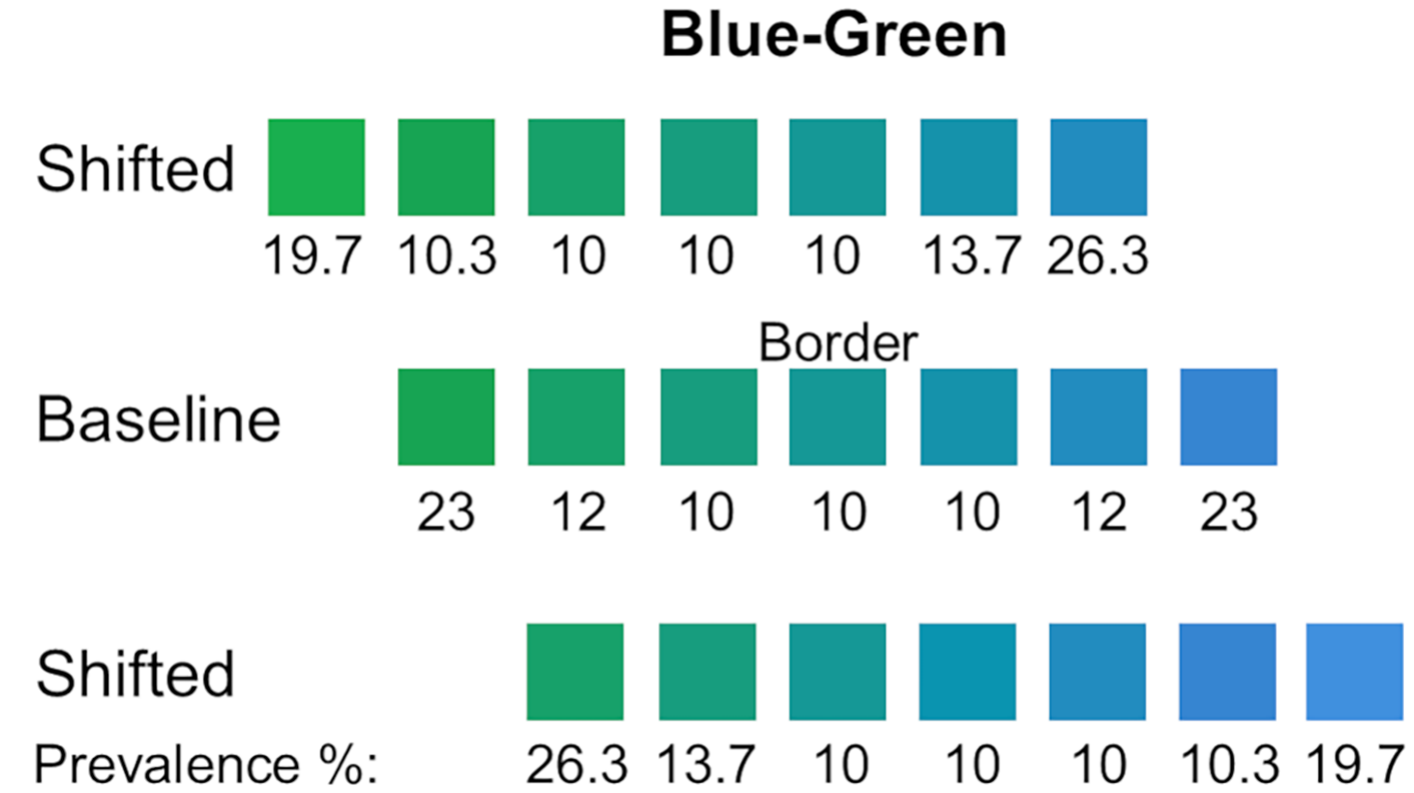
The three sets of stimuli tested are shown, with the prevalence (frequency of presentation) for each hue indicated below the respective rows. The chromaticities of these stimuli are identical to those used in Experiment 1 for both the blue-green and pink-purple boundaries (see Figure 2 for CIE coordinates). This prevalence scheme was designed to equalize the proportion of stimuli from each category and the subsequent proportion of responses made with each saber color, thereby isolating the effect of stimulus range from response frequency.

The same set of green-blue and pink-purple hues was used as in the first experiment. However, in this study, the prevalence of hues was adjusted to ensure an equal proportion from each color category. Figure 6 illustrates the hue distribution for the blue-green range. The baseline condition mirrored the first experiment, with hues equally distributed on either side of the category border. In the green-shifted condition, more blue hues were presented, whereas in the blue-shifted condition, more green hues were shown. This ensures the proportion of responses made with each saber color is equalized. The distribution of green and blue hues was adjusted for the two prototypical hues at the endpoints for each category in each set of hues. Because the middle three hues are ambiguous, they stayed at a constant prevalence of 10%. The same prevalence distribution was used for pink-purple hues which were tested as well.

### Results

**Figure 7.**
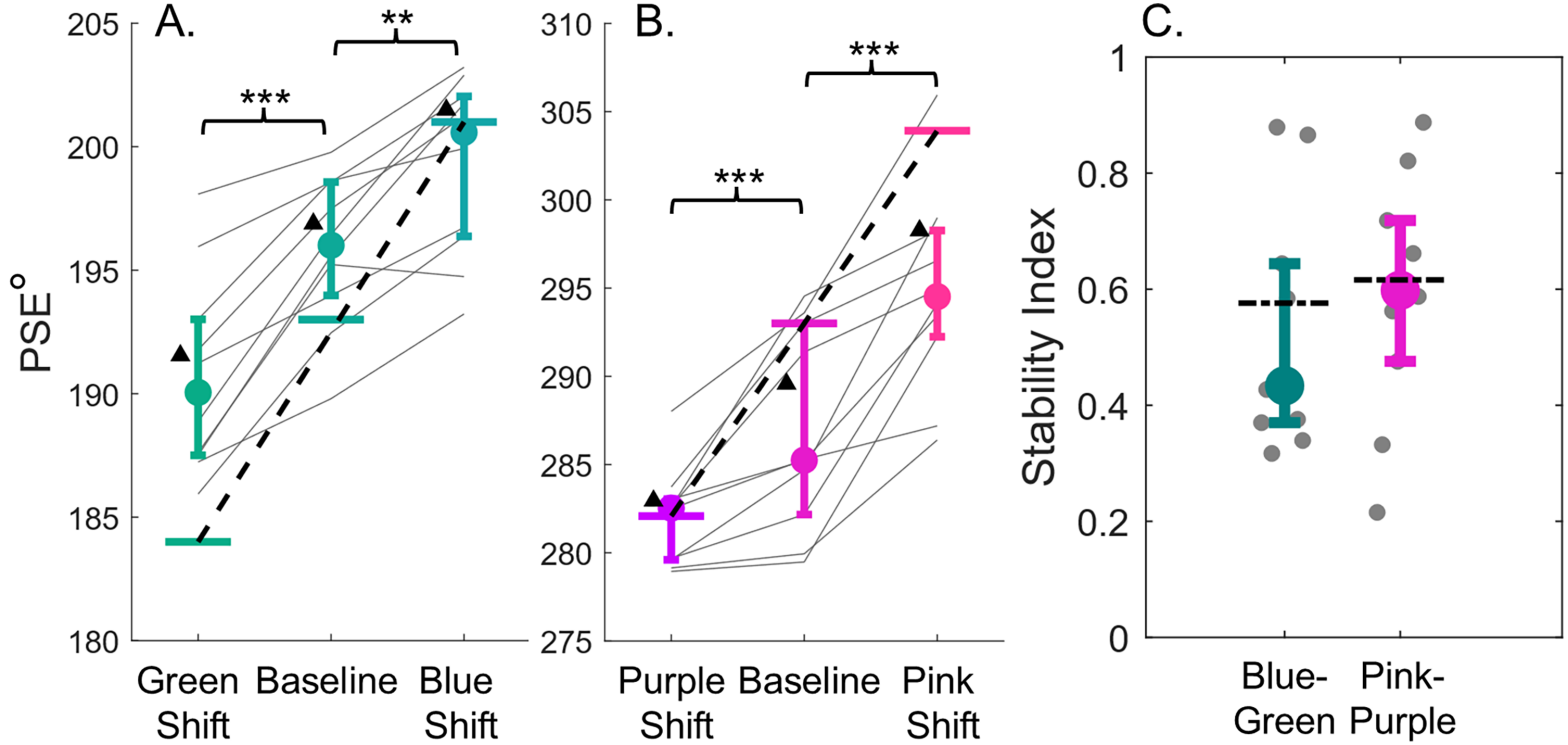
The median PSE for blue-green (**A**) and pink-purple (**B**) hues. The PSE represents the color category border. Data from the first experiment's PSEs are shown as black triangles (**C**). The median stability index, where a value of 0.0 would correspond to stable color borders. The dotted black line shows the stability index from the first experiment for the blue-green and pink-purple sessions. Error bars show the interquartile range.

Our goal in this follow-up study is to determine whether the partial shift effect shown in the first experiment persists when the proportion of responses made with each saber is equalized. We found that compared to the first experiment, the proportion of swipes made with each saber was consistent, and close to 50% for each saber hue. As in the first experiment, we computed psychometric fits and the PSE for the data from each condition tested. The PSE, representing the color category border, is shown in Figures 7A and 7B for blue-green and pink-purple hues.

The data indicates a similar pattern to the first experiment; color category borders are shifted in the direction of the saber color shift. A one-way ANOVA revealed significant main effects for green-blue [*F*(2, 16) = 47.0, *p* < 0.001, $\eta_{p}^{2}$ = 0.86] and pink-purple [*F*(2, 16) = 37.82, *p* < 0.001, $\eta_{p}^{2}$ = 0.83] PSEs, with post-hoc tests revealing significant differences between all conditions (all *p* < 0.004).

The current experiment shows color category borders shifting in alignment with the shift in tested hues, and the saber colors. What is the magnitude of the category border shift, as compared to the hue/saber shifts? Figure 7C shows the stability index between the PSEs and the hues tested for the shifted conditions. The results of the current experiment show that as opposed to a range effect or stable borders, the data again fall between stable category borders and a full shift of the PSEs consistent with a range effect. The difference in the category borders falls short of a full range effect, but is inconsistent with stable category borders. This finding aligns with the results from our first experiment.

### Summary

Our results indicate that color category borders are dynamically influenced by the statistics of the stimulus set. Specifically, color category borders shift with the underlying distribution of hues tested. The observed shift, although partial rather than absolute, is indicative of a partial adaptation. This outcome is distinct from a complete shift, which would arise if responses were based solely on the statistics of the underlying stimulus distribution. Crucially, this partial range effect is resilient to manipulations in the prevalence of the tested hues, remaining robust even when the proportion of swipe responses made with each saber color was equalized. This highlights that the observed bias is a statistical adaptation rather than a simple response bias.

In both studies the saber color hues shifted with each block, and were also included as stimuli in each block. As a result, for some discriminations, the hue tested perfectly matched one of the sabers. There are two possible interpretations of these findings. The first is that the similarity of the stimulus hue to the sabers is driving this effect. As the saber colors shift with the shift in hues tested, such a similarity effect could drive the pattern of results seen. The other possibility is that observers adapt to the underlying distribution of stimuli tested, and shift their category border with respect to the distribution of stimuli presented—a range effect.

Experiment 3 was designed to dissociate these explanations. Here, the saber colors were held constant across all blocks and were never included in the test sets. If the partial border shifts observed in Experiments 1 and 2 were driven by direct similarity between cube and saber hues, eliminating perfect matches should substantially reduce or eliminate the effect. Conversely, if observers truly adapt to the range of presented hues, the partial shift should persist despite the disruption of stimulus–saber similarity.

## Experiment 3: Control for the similarity effect

The following experiment uses the same paradigm and hues as in the previous studies, but introduced a key manipulation: the saber hues remained constant between experimental blocks, and were distinct from the test hues. This manipulation not only shifts the range of hues tested as in prior work, but also disrupts the similarity between the saber and test hues, because the two are never identical.

### Methods

#### Observers

Twenty new observers (14 female, 6 male, aged 19-33) participated. All were naive to the purpose of the experiment and had normal color vision as confirmed by the Ishihara color vision test (Ishihara, 1997). The experiment followed the same design, apparatus, and stimuli as the first experiment, with exceptions noted below.

#### Experimental design

**Figure 8.**
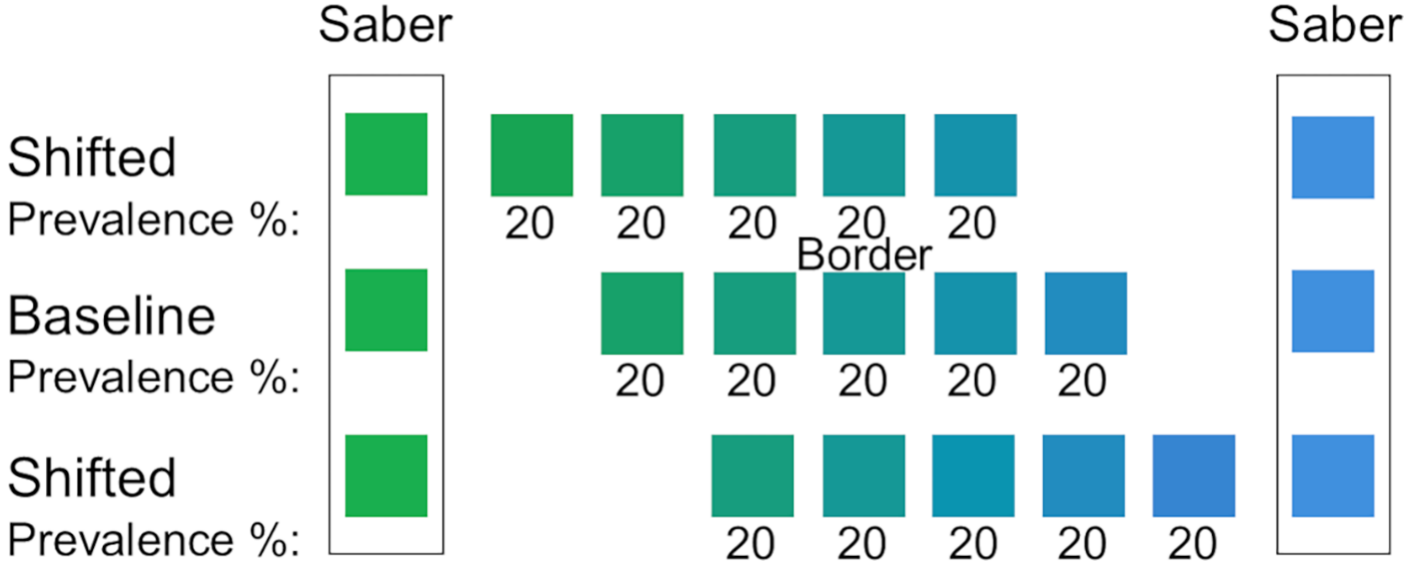
The hues tested in the current experiment. Note the saber colors remained constant across the three blocks, and that the prevalence of each hue within a block remained consistent at 20%.

This experiment focused on blue-green hues, including a category border. The hues were the same as those used in earlier studies. However, the saber colors—one blue and one green—remained constant across all three blocks. To ensure the saber colors were not included in the test set, we limited each block to five test hues. One block served as the “baseline,” estimating the category border as the central hue, whereas the other blocks shifted the hues toward green or blue. Each hue appeared with equal frequency (20%). A schematic of the tested hues and saber colors is provided in Figure 8.

### Results

**Figure 9.**
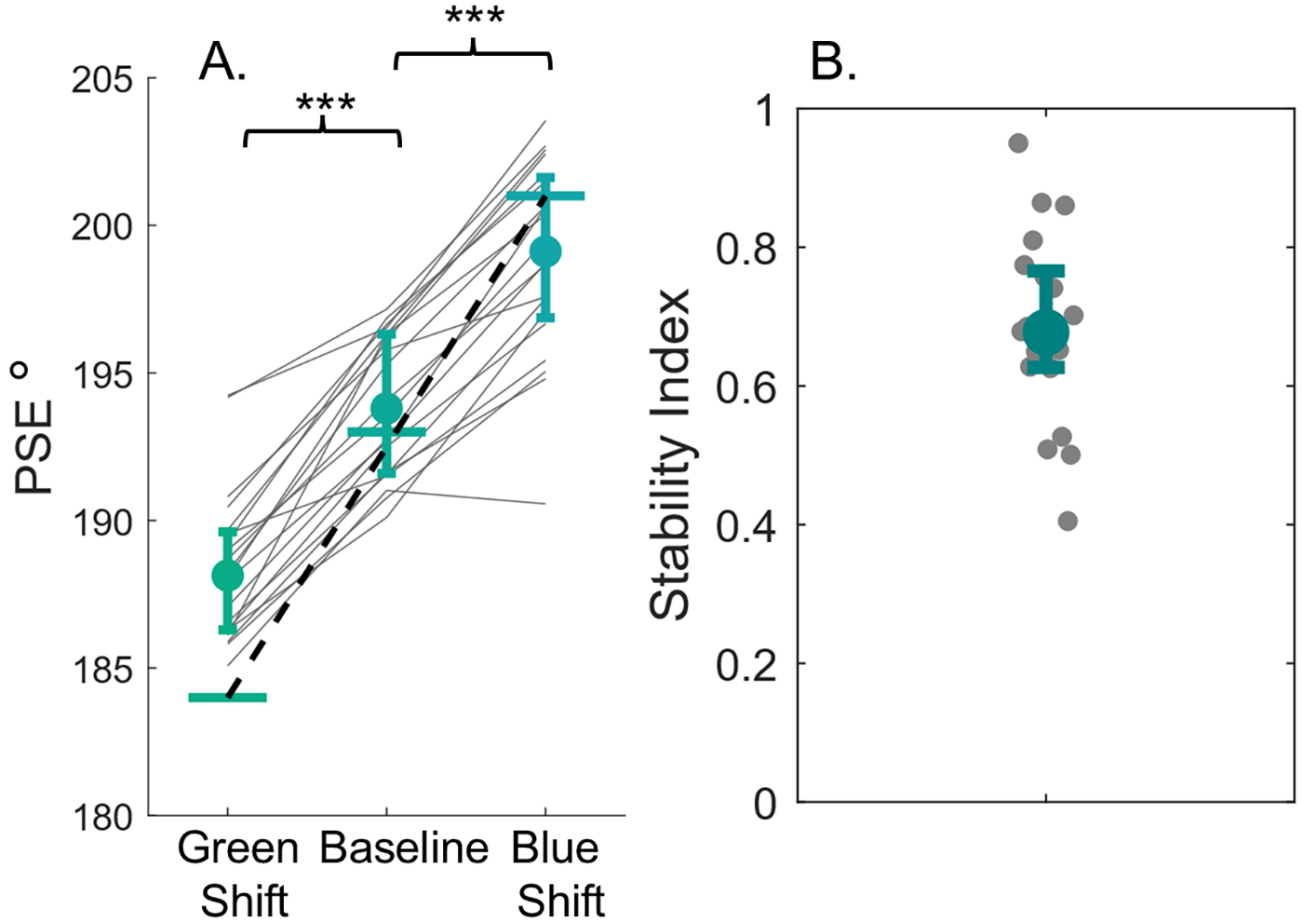
(**A**) Average PSE for blue-green hues. The circular markers show estimates for the blue-green category border. (**B**) The stability index for blue green hues. Error bars show the interquartile range.

This follow-up experiment aimed to test whether the similarity between saber and test hues influences the stability of color category borders. By excluding the saber colors from the test hues, we aimed to discourage observers from relying on a similarity-based matching strategy. As in previous experiments, we computed psychometric fits and the PSE for each condition to estimate the color category border. Figure 9A shows the average PSEs revealing a pattern of shifts similar to the previous experiments. A one-way ANOVA shows a significant main effect of PSE [*F*(2, 38) = 237.4, *p* < 0.001, $\eta_{p}^{2}$ = 0.70] with post-hoc tests indicating significant differences between all conditions (*p* < 0.001). The direction of the shift in PSEs is similar to previous studies, but is the magnitude of the shift in PSEs comparable? Figure 9B illustrates the stability index between PSEs and the tested hues for the shifted conditions. The results suggest that, rather than a full similarity effect, the data fell between stable category borders and a full range effect, consistent with previous findings.

### Summary

Experiment 3 addressed the possibility that the partial border shifts observed in the earlier experiments were driven by a similarity-based strategy (i.e., by observers comparing the hue of each cube to the saber colors rather than adapting to the range of presented hues). Because the saber colors shifted with the stimulus range in Experiments 1 and 2, a simple similarity rule could mimic a range effect. By keeping the saber colors constant across blocks and excluding these hues from the test sets, Experiment 3 eliminated any perfect matches between stimuli and sabers.

Despite this manipulation, participants continued to show robust partial shifts in their category borders in the direction of the stimulus-range shift. Thus removing the possibility of perfect hue matches did not eliminate or weaken the effect. This strongly argues against a similarity-based explanation and supports the interpretation that observers adapt to the distribution of tested hues—a partial range effect—rather than relying on a simple perceptual comparison with the sabers.

Up to this point, however, all experiments included hues that straddled an estimated color category border, such as the blue–green or pink–purple divide. One remaining question is whether the presence of a category boundary itself constrains the shift. A category border might act as a perceptual anchor, limiting the degree to which the PSE can move; alternatively, the partial shift might arise even when no linguistic or perceptual boundary is present. To distinguish between these possibilities, Experiment 4 tested a set of green hues without any category border, allowing us to assess whether partial adaptation persists in the absence of categorical structure.

## Experiment 4: Green hues: No category border

Experiment 4 examined whether the presence of a color category boundary influences the magnitude of the PSE shift. Using the same VR paradigm and DKL-defined hue sets as in previous experiments, we presented participants with a series of green hues that lie within a single color category in English and do not cross any known category boundary. If a category border stabilizes the decision boundary, its absence should increase the magnitude of the shift. Conversely, if partial adaptation reflects a general statistical normalization process, then the PSE should shift partially toward the center of the tested hue range even when no category boundary is present.

### Methods

#### Observers

Ten new observers (eight female, two male, aged 22–31) participated. All were naive to the experiment's purpose and had normal color vision (Ishihara, 1997).

#### Hue selection

**Figure 10.**
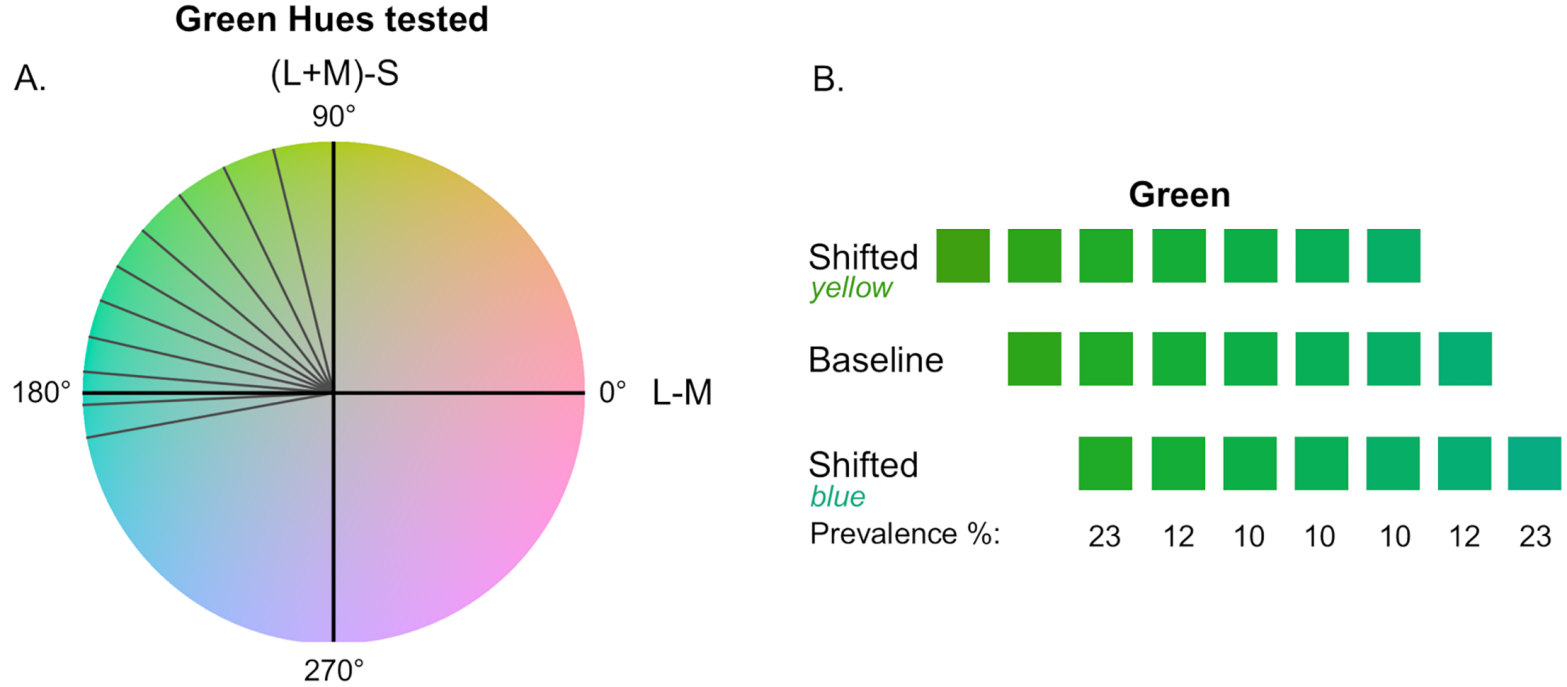
(**A**) The isoluminant plane of the DKL color space. The gray lines show the hue angles of the colors selected for the present study. 1.2 JNDs separate each of these lines. (**B**) The three sets of hues tested. Hues were shifted towards yellow and blue relative to the baseline in the shifted conditions. There are 1.2 JNDs between neighboring hues.

Nine green hues were selected for testing. The green category was selected for testing as it broadly spans DKL color space; no other hue category spans a large enough area to test without encroaching on neighboring categories. These hues are shown in Figure 10A, with stimulus details in Supplementary Appendix A. As in the previous experiments, hues were selected based on discrimination threshold curves from Witzel and Gegenfurtner, (2013) ensuring 1.2 JNDs between adjacent hues. We used the selected hues to generate sets of green hues shifted towards yellow and shifted towards blue, as shown in Figure 10B.

#### Experiment design

The apparatus and setup were identical to the previous studies. Each observer completed three blocks: a baseline session with green hues and two sessions with shifted hues (one toward blue and the other toward yellow). Each block lasted 16 minutes, and the block order was counterbalanced using a Latin square design.

### Results

**Figure 11.**
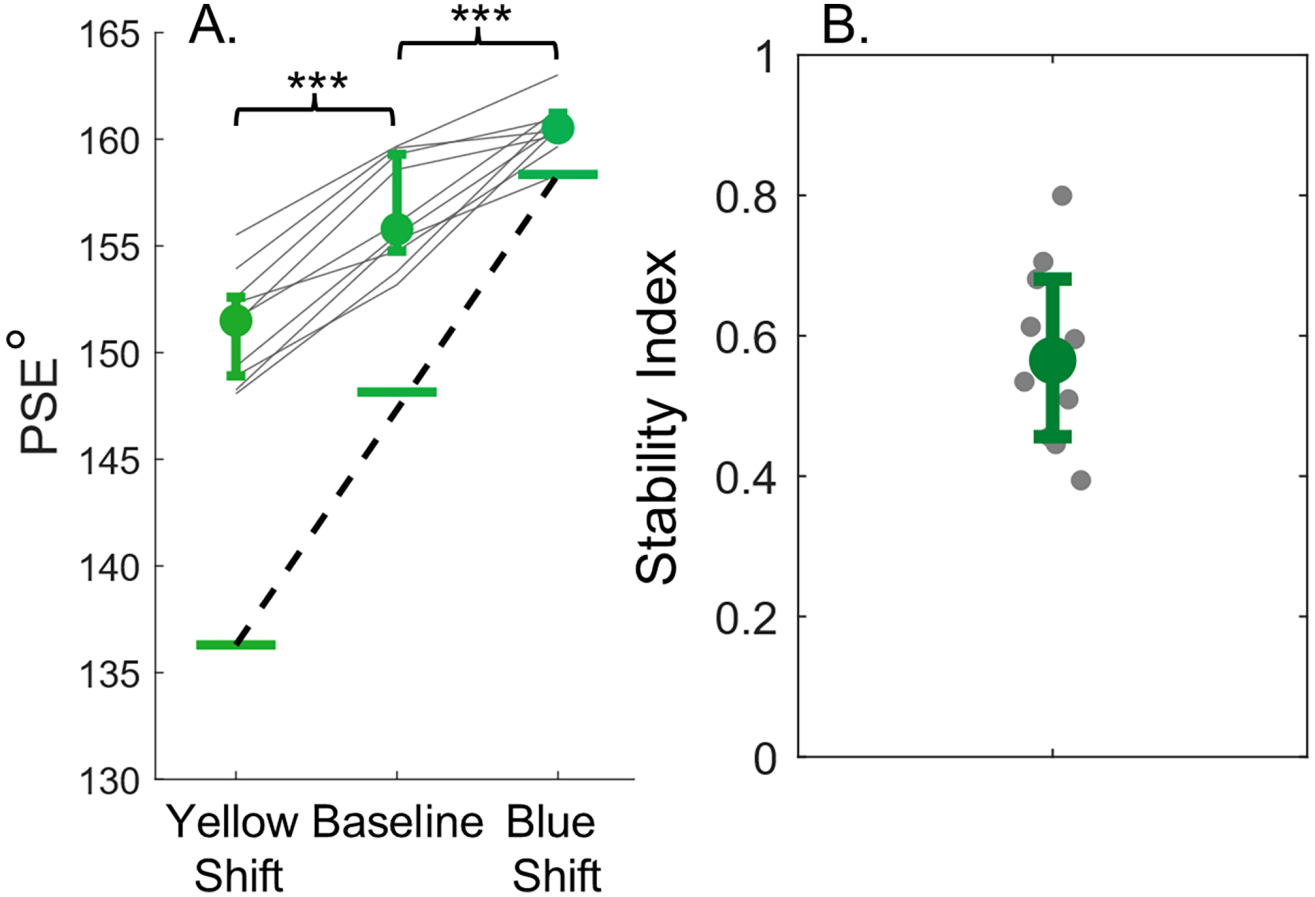
(**A**) Median PSE for green hues. These hues did not include a color category boundary, and the PSEs represent the decision boundary. (**B**) Stability Index for green hues. All error bars show interquartile range.

We computed psychometric functions for each block of trials to investigate the stability of the decision boundary (Psignifit toolbox; Schütt et al. (2016)). Figure 11A displays the average PSE across observers. The PSEs are noticeably displaced in the shifted conditions relative to the baseline condition, indicating the decision boundary shifted with the range of hues tested. An ANOVA revealed a significant main effect of condition [*F*(2, 18) = 109.25, *p* < 0.001, $\eta_{p}^{2}$ = 0.92]. Post-hoc tests indicate significant differences between PSEs for all conditions (all *p* < 0.001).

Figure 11B presents the stability index, which quantifies the PSE stability in the shifted conditions compared to the tested hues. The results were consistent with earlier experiments, which featured hues that included a color category border. The data show shifts to the decision boundary that fall between a stable decision boundary and a full adaptation to the range of hues tested.

### Summary

Experiment 4 tested sets of green hues that did not include a color category boundary, using the same design as in the previous experiments. The results again showed that the estimated decision boundary shifted in the direction of the tested hue range. As before, the shift was intermediate between a fully stable boundary and the full displacement predicted by a similarity-based strategy.

Crucially, this pattern emerged even in the absence of a category border, demonstrating that the partial adaptation effect does not depend on crossing a linguistic or perceptual category boundary. Instead, the decision boundary appears to adjust to the statistical distribution of the presented hues, indicating that category structure does not moderate the shift effect observed across all experiments.

## Discussion

Our study adapted a paradigm traditionally used in animal learning to examine the stability of human color category borders. Using an immersive VR environment, we developed a fast, engaging task that allowed us to test how decision boundaries shift when the distribution of presented hues is manipulated. Across experiments, we found that the estimated category border shifted in the direction of the change in the hue range, for both the blue–green and the pink–purple regions. If category borders were stable, no such differences would have emerged; conversely, a pure similarity strategy based on the distance between cube and saber hues would have produced a full shift matching the stimulus displacement. Instead, observers consistently showed a partial shift.

This partial shift persisted when the similarity between cube and saber hues was eliminated as a cue, when the prevalence of hues from each category was equalized, and even when the tested hues did not include a category boundary at all. Together, these results indicate that color category borders are less stable than often assumed and that categorical structure played only a limited role in this task. Rather than reflecting fixed linguistic boundaries or simple hue-similarity matching, the decision boundary appears to adapt to the statistical distribution of the presented hues—a partial range effect.

### Mechanisms underlying the partial shift

The consistent partial shift of the category border across Experiments 1–3 provides strong constraints on the underlying mechanism. Although these shifts were too large to be compatible with a strictly fixed, linguistically defined boundary, they might still be reconciled with certain forms of categorical perception. In principle, observers might maintain a stable internal category boundary but adjust their decisions around that boundary due to decisional factors, task demands, or the salience of particular exemplars. Under such an account, the observed partial shifts would reflect flexible decision criteria within a categorical framework rather than adaptation of the boundary itself.

However, Experiment 4 rules out categorical-boundary-driven mechanisms as the primary explanation. When observers classified a set of green hues that did not contain any category boundary—neither linguistic nor perceptual—the same partial shift was observed. With no category structure available, the shift cannot be attributed to categorically anchored decision processes. This finding demonstrates that categorical perception alone cannot explain the pattern of results and that the mechanism underlying the shifts must operate independently of category boundaries.

A more parsimonious explanation is that observers engage in statistical adaptation to the distribution of presented hues. Under this account, the decision boundary reflects a weighted combination of the sensory signal and an implicit prior centered on the mean of the tested range. Such a mechanism fits naturally within Bayesian models of perceptual estimation, where judgments arise from integrating variable sensory information with expectations about the stimulus distribution (Ashourian & Loewenstein, 2011; Jazayeri & Shadlen, 2010). Partial adaptation emerges when neither the sensory evidence nor the prior dominates, producing shifts intermediate between a fully stable boundary and a full similarity-based shift.

Importantly, this mechanism does not rely on low-level chromatic structure or stimulus–response similarity. The similarity of the effects across the blue–green and pink–purple regions shows that the shifts are not tied to cone-opponent nonlinearities. The survival of the shift in Experiment 3—where perfect hue matches between sabers and test stimuli were eliminated—demonstrates that the mechanism is not a simple hue-comparison strategy. And Experiment 4 conclusively shows that the mechanism does not require any categorical structure at all. Together, these results indicate that the partial shift reflects a general normalization process, driven by the statistical distribution of the stimuli rather than by categorical anchors, perceptual boundaries, or similarity judgments.

### Relation to prior work

#### Categorical perception

Classical studies of categorical color perception showed enhanced discrimination across category boundaries and linguistic modulation of such effects (Bornstein & Korda, 1984; Gilbert et al., 2006; Holmes et al., 2009; Witzel & Gegenfurtner, 2011; Thierry et al., 2009; Winawer et al., 2007). Training studies likewise demonstrated that newly learned boundaries can alter discrimination (Özgen, 2004; Özgen & Davies, 2002; Zhou et al., 2010). Within this framework, category borders have often been treated as relatively stable perceptual structures.

Experiments 1–3 in the present study could, in principle, still be interpreted within such a categorical framework: observers might maintain a stable internal boundary while adjusting their decisional criteria around it. However, Experiment 4 strongly argues against a category based explanation. When observers judged only green hues—without any linguistic or perceptual category boundary—the same partial shift emerged. Thus the bias is not dependent on the presence of a category boundary and cannot be explained by categorical anchoring alone.

#### Relation to range effects and statistical adaptation

A separate line of work emphasizes that perceptual judgments are drawn toward the mean or statistical center of recently encountered stimuli. Such range effects, well documented across many domains (Hollingworth, 1910; Huttenlocher et al., 2000; Jazayeri & Shadlen, 2010; Ashourian & Loewenstein, 2011; Olkkonen et al., 2014), have recently been demonstrated for color as well. Saarela et al. (2023) showed that both range effects and serial dependence bias estimates of hue. However, their task examined memory-based or perceptual estimates of individual hues, not the stability of the category boundary itself.

Our study makes a novel contribution by showing that the range effect is strong enough to shift the category border—that is, the hue at which observers are equally likely to classify a stimulus as belonging to one category or another. This addresses a question left open by prior work: whether the color category boundary is an invariant semantic structure or a flexible, adaptively drawn line. The presence of a partial shift, even when no category boundary is present (Experiment 4), indicates that the border behaves as a statistical decision boundary rather than a fixed categorical anchor.

Our manipulation also differs from Saarela et al. (2023) in that we explicitly and consistently controlled the mean and range of the stimulus distribution across blocks. Whereas Saarela's CTB emerged indirectly from a staircase, our paradigm systematically shifted the distribution itself, allowing us to show that the category border changes in a predictable, lawful manner relative to the manipulated range. This provides more direct evidence that categorization is a flexible, adaptive process influenced by the stimulus statistics.

Virtanen et al. (2024) reported that ensemble color judgments depend only weakly on category membership, suggesting that summary color perception is largely independent of linguistic categories. Our findings complement and extend this conclusion by showing that decision boundaries themselves are not fixed: they adapt to the statistical structure of the input even in a discrete, forced-choice categorization task. This suggests that categorical labels play a limited role in shaping perceptual decisions under dynamic, rapidly changing conditions.

#### Controlling for low-level sensory mechanisms

A further contribution stems from the fact that much prior work—including both categorical-perception studies and range-effect research—has focused disproportionately on the blue–green boundary, which coincides with the L–M cone-opponent axis. This raises the possibility that some reported effects reflect low-level retinal mechanisms rather than categorical or statistical processes.

By defining our stimuli in JND units (Witzel & Gegenfurtner, 2013) and including the pink–purple axis, which lies outside the cardinal opponent directions, we were able to test whether sensitivity variations explain the observed pattern. The persistence of the partial shift in the pink–purple region demonstrates that the effect is not tied to cone-opponent nonlinearities. Experiment 4 further shows that neither category boundaries nor cardinal axes are necessary for the bias to emerge.

### The role of training, task experience, and active engagement

A further consideration is whether task experience influences the extent to which observers rely on categorical distinctions. Previous work has shown that categorical facilitation is strongest in naïve observers and diminishes with training or repeated exposure (Witzel & Gegenfurtner, 2015). In our paradigm, observers made rapid, repeated decisions, and may therefore have learned early in the task to rely on direct hue comparisons rather than on linguistic category labels. This interpretation aligns with accounts of “fast psychophysics,” in which rapid feed-forward visual processing supports efficient decisions without explicit categorical evaluation (Schmidt et al., 2011; Lamme & Roelfsema, 2000).

Task difficulty or perceptual sensitivity cannot explain the observed shifts. Although discrimination thresholds (σ) varied across observers and experiments, this variability did not predict the magnitude of the category-border shift, nor did it correlate with range-effect strength, consistent with earlier findings by Olkkonen et al. (2014).

In addition, the active, immersive nature of our VR paradigm may influence perceptual processing. Unlike traditional, static psychophysical tasks in which observers remain stationary and perform deliberative judgments, our participants continuously moved, oriented toward incoming stimuli, and executed rapid motor responses. Movement is known to modulate visual cortical state: in rodents, locomotion increases firing rates and improves stimulus encoding in early visual cortex by reducing noise correlations (Niell & Stryker, 2010; Dadarlat & Stryker, 2017). Although the specific mechanisms may differ in humans, these studies suggest that active engagement can enhance the efficiency of sensory processing. It is therefore plausible that the dynamic, action-based setting of our task encouraged observers to rely primarily on immediate perceptual cues rather than on categorical distinctions that require slower, attention-dependent processing.

Taken together, these considerations suggest that with experience and continuous action, observers may shift toward rapid hue-based decisions and away from categorical strategies. This helps explain why color categories played only a limited role in our task and why partial adaptation to the stimulus distribution emerged even when no color category boundary was present.

### Stability, partial shift, and full shift

A central feature of our findings is that the decision boundary shifted partially, rather than fully, toward the mean of the tested hue range. This intermediate pattern rules out both the idea of a fixed, invariant color category boundary and the possibility that observers rely exclusively on hue similarity between the test and saber colors. Instead, a partial shift suggests that observers combine multiple sources of information when making categorical judgments.

A compelling account arises from a Bayesian or statistical-normalization framework, in which observers integrate the current sensory signal with expectations based on the distribution of encountered stimuli (Ashourian & Loewenstein, 2011; Jazayeri & Shadlen, 2010). Under such models, perceptual decisions are influenced by both immediate sensory evidence and a prior centered on the stimulus distribution. Because sensory encoding of hue is noisy, the prior cannot fully override the perceptual input, and the perceptual signal cannot be interpreted independently of context. This naturally yields an intermediate decision boundary, similar to the partial range adaptation observed in our experiments.

Individual differences reinforce this interpretation. While all observers showed partial shifts, the magnitude varied across individuals. This pattern is consistent with differences in the relative weighting of priors and sensory uncertainty, a principle observed in other perceptual domains (Webster, 2020; Simoncelli & Webster, 2024). Observers with more precise long-term perceptual anchors or lower sensory noise may show smaller shifts, whereas those with weaker or less stable anchors may show larger ones. Despite this variation, no observer exhibited a fully stable or fully shifted boundary, indicating that partial adaptation reflects a robust, general mechanism.

A further factor that may constrain the size of the shift is the presence of appearance-based perceptual anchors, such as the “unique” hues. In Experiment 4, where no category boundary was present, the baseline PSE for green tended to fall between the greenish and yellowish side of the tested range. One possibility is that observers implicitly referenced a perceptual anchor close to unique green, which is known to be judged with high precision (Hering, 1920; Valberg, 2001; Webster, 2020). If unique green acts as a stable internal reference point, it would resist complete displacement even when the tested hue distribution is skewed. Such an anchor would not depend on linguistic categories and could help explain why the shift remains partial in both categorically defined and category-free hue ranges. The existence of such internal reference points could also contribute to the observed individual differences.

Task-specific constraints offer an additional explanation for partial rather than full adaptation. Our immersive VR paradigm requires rapid, continuous decisions under time pressure. These conditions favor fast, feed-forward perceptual processing rather than slow, attentionally mediated evaluation of category structure. As in many classical range-effect studies (Hollingworth, 1910; Parducci, 1965; Olkkonen et al., 2014), observers seldom recalibrate entirely to the stimulus mean; instead, they adjust in a graded fashion while retaining influence from stable perceptual priors.

Together, these considerations show why the shift is neither zero nor complete. The decision boundary reflects a weighted integration of sensory input, task demands, stimulus statistics, and stable perceptual anchors such as unique hues or category borders. The partial nature of the shift therefore provides a strong constraint on mechanistic interpretations, contradicting fixed-category and pure-similarity models while supporting a flexible, context-sensitive decision process grounded in statistical adaptation.

Although our results demonstrate a consistent range effect across participants, the current study was not designed to assess how individual differences—such as linguistic background, sex, or specific adaptation states—might modulate the magnitude of this shift. Future research using larger, heterogeneous cohorts would be valuable for exploring these individual variances.

### Conclusions and outlook

The present findings carry several implications for theories of color perception and categorical decision-making. First, they suggest that color categories may play a more limited role in rapid perceptual judgments than previously believed. In immersive or action-driven settings, observers appear to rely primarily on immediate sensory information and the statistical distribution of stimuli rather than on fixed category labels. This raises the possibility that many classical categorical-perception effects may emerge only under tasks that encourage slower, more deliberate evaluation of color differences.

Second, our results demonstrate that category borders are not tied to specific regions of color space, such as the blue–green boundary, nor constrained by early cone-opponent mechanisms. The presence of a similar partial adaptation in the pink–purple region and in a category-free green hue set suggests a general normalization process rather than one tied to linguistic or physiological structure. This opens the door for future work to investigate how decision boundaries are formed in more complex or naturalistic color distributions, including those involving illuminant variation or material cues.

Third, the robust partial shift across observers highlights the role of perceptual anchors, such as unique hues, and the balance between long-term priors and short-term statistical adaptation. Future studies could examine how these anchors vary across individuals and cultures, and how they interact with stimulus statistics during rapid decision-making.

Methodologically, the VR paradigm introduced here shows promise as a tool for high-throughput measurement of perceptual decision boundaries. Its dynamic, engaging format makes it well suited for studying detection and discrimination, adaptation, learning, and perceptual stability under more ecologically valid conditions.

Finally, these results invite a re-examination of the relationship between linguistic categories and perceptual decisions. If color category borders can shift within minutes in response to stimulus statistics, then their stability in everyday settings may depend on long-term environmental regularities and communicative demands rather than immutable perceptual structure. Cross-linguistic and developmental studies using similar rapid-decision paradigms may help illuminate how stable categorical distinctions emerge despite this underlying flexibility.

## Supplementary Material

Supplement 1
