# Supplementary material for: Swiping colors in virtual reality: How stable are color category borders?
Source: J Vis. 2026 Apr 16;26(4):9. doi: 10.1167/jov.26.4.9 (PMC13101840; doi:10.1167/jov.26.4.9)

Appendix

Appendix A.

Hues Tested	Hue Angle	x	y
blue-green	158	0.21	0.59
blue-green	167	0.20	0.53
blue-green	176	0.20	0.48
blue-green	184	0.19	0.42
blue-green	193	0.18	0.37
blue-green	201	0.18	0.31
blue-green	210	0.18	0.27
blue-green	221	0.17	0.23
blue-green	232	0.18	0.19
pink-purple	255	0.19	0.15
pink-purple	264	0.21	0.14
pink-purple	272	0.22	0.14
pink-purple	282	0.24	0.15
pink-purple	293	0.27	0.15
pink-purple	303	0.3	0.17
pink-purple	317	0.34	0.19
pink-purple	332	0.40	0.22
pink-purple	348	0.45	0.26

Table 1. CIE 1931 xy and DKL hue angle values for the blue-green and pink-purple colors tested.

Hues tested	Hue Angle	x	y
green	115	0.24	0.63
green	125	0.18	0.65
green	135	0.12	0.67
green	145	0.08	0.68
green	153	0.05	0.69
green	161	0.03	0.69
green	168	0.02	0.69
green	175	0.02	0.68
green	182	0.03	0.68

Table 2. CIE 1931 xy and DKL hue angle values for the colors tested in the green hues experiment.

Appendix B.

Figure 1. σ, a parameter from the psychometric fits of the cumulative Gaussian function, represents discrimination performance, with lower values indicating better performance. Each plot shows the average σ for both the baseline and shifted conditions, with error bars representing the interquartile range. Panels A and B present data from the current experiment. Panels C and D display results from a second study that tested a balanced prevalence of stimuli. Panel E shows results from a third control experiment investigating whether a similarity effect caused the PSE shifts. Finally, Panel F presents data from a fourth experiment testing green hues, without a category border.

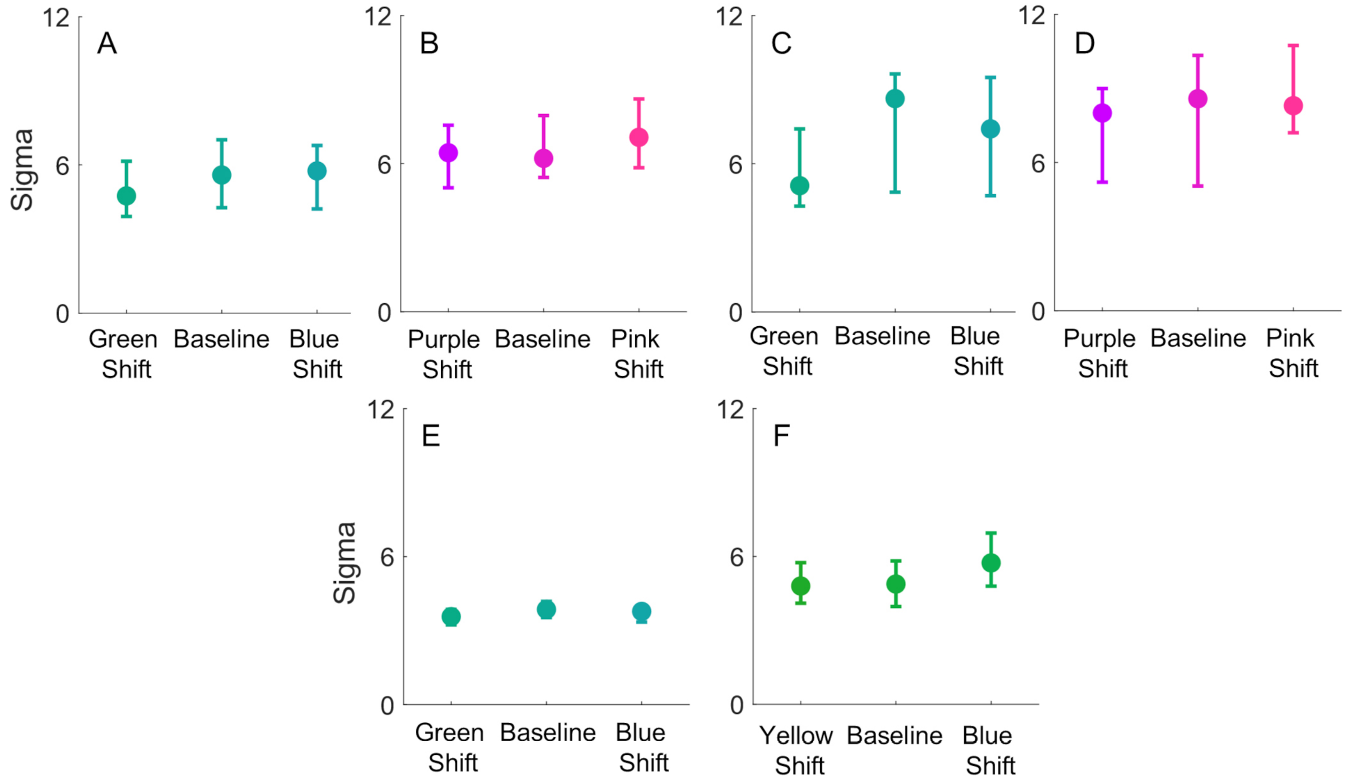

Supplement: Supplement 1 [file jovi-26-4-9_s001.pdf]
